# Low-Molecular-Weight Chondroitin Sulfates Alleviate Simulated Microgravity-Induced Oxidative Stress and Bone Loss in Mice

**DOI:** 10.3390/cimb45050268

**Published:** 2023-05-10

**Authors:** Rong Lan, Ye Li, Xinying Zhao, Rong Shen, Ruili Wang, Ruixin Mao, Shuangsheng Guo

**Affiliations:** 1 Beijing Polytechnic Institute, College of Bioengineering, Beijing 100176, China; lanrong@bpi.edu.cn (R.L.); zhaoxinying@bpi.edu.cn (X.Z.); shenrong@bpi.edu.cn (R.S.); rlwang16@iccas.ac.cn (R.W.); 2 Department of Environmental Control and Life Support System, China Astronaut Research and Training Center, Beijing 100094, China; mrxsyzf@163.com

**Keywords:** simulated microgravity, low-molecular-chondroitin sulfate, hindlimb suspension, anti-oxidative stress, anti-bone loss

## Abstract

(1) Background: Many studies have shown that microgravity experienced by astronauts or long-term bedridden patients results in increased oxidative stress and bone loss. Low-molecular-weight chondroitin sulfates (LMWCSs) prepared from intact chondroitin sulfate (CS) have been demonstrated to possess good antioxidant and osteogenic activities in vitro. This study aimed to assess the antioxidant activity of the LMWCSs in vivo and evaluate their potential in preventing microgravity-induced bone loss. (2) Methods: we used hind limb suspension (HLS) mice to simulate microgravity in vivo. We investigated the effects of LMWCSs against oxidative stress damage and bone loss in HLS mice and compared the findings with those of CS and a non-treatment group. (3) Results: LMWCSs reduced the HLS-induced oxidative stress level, prevented HLS-induced alterations in bone microstructure and mechanical strength, and reversed changes in bone metabolism indicators in HLS mice. Additionally, LMWCSs downregulated the mRNA expression levels of antioxidant enzyme- and osteogenic-related genes in HLS mice. The results showed that overall effect of LMWCSs was better than that of CS. (4) Conclusions: LMWCSs protect against the bone loss caused by simulated microgravity, which may be related to their ability to reduce oxidative stress. LMWCSs can be envisaged as potential antioxidants and bone loss protective agents in microgravity.

## 1. Introduction

The microgravity environment experienced by astronauts performing space missions or long-term bedridden patients can cause physiological and pathological changes, such as osteoporosis and cardiovascular dysfunction, which are closely related to oxidative stress injury [1,2]. Therefore, developing effective protective strategies against oxidative stress damage in microgravity is essential. Currently, the countermeasures taken against microgravity-induced oxidative stress are exercise, nutritional supplementation, and medication. However, exercise and nutritional supplementation alone are not sufficient to prevent the pathophysiological changes caused by microgravity [3,4]. Therefore, there has recently been increasing interest in finding drugs which will combat the oxidative stress damage and pathophysiological changes induced by microgravity. In recent years, some natural products with antioxidant effects have achieved initial success in the prevention and treatment of these changes [5,6,7], but the number of studies remains small. Therefore, antioxidant drugs that are effective at preventing and treating pathologies under microgravity conditions need to be urgently developed.

Chondroitin sulfate (CS) is a type of sulfated glycosaminoglycan composed of alternating D-glucuronic acid and N-acetyl-D-galactosamine units [8]. It is mainly found in cartilage, bone, tendons, sarcolemma, and blood vessel walls in humans and animals. CS has significant antioxidant activity [9,10,11], as well as anti-inflammatory [12], immunomodulatory [13], cardiovascular- and cerebrovascular-protective [14], neuroprotective [15], and other pharmacological effects. Currently, it is widely used as a safe and effective anti-osteoarthritis drug and as functional food [16,17]. It is reported that low-molecular-weight chondroitin sulfates (LMWCSs) produced by the degradation of CS have better biological activities than intact CS [18,19]. Recently, we prepared LMWCSs from intact CS using a complex enzyme hydrolysis process. We previously revealed that the antioxidant activities of the LMWCSs in vitro were superior to those of CS. Furthermore, within the concentration range of 25–200 μg/mL, the proliferation activities of LMWCSs on osteoblasts were better than those of CS [20]. Therefore, the LMWCSs prepared from CS may become active ingredients in antioxidant drugs and healthy foods and have the potential to develop protectants for bone loss. However, more studies need to be performed to confirm their antioxidant potential and to assess their potential for preventing bone loss in microgravity conditions.

In this paper, we studied the antioxidant activity of LMWCSs in vivo, and explored their protective effect and mechanism of action on oxidative stress damage and bone loss. Simultaneously, the differences in the antioxidant activities and protective effects of LMWCSs and CS were assessed. We hypothesized that LMWCSs have good antioxidant activity and an anti-bone loss effect in vivo, and that their anti-bone loss effect might be related to the reduction of oxidative stress in mice. To test this hypothesis, we studied the effects of LMWCSs on bone tissue and serum oxidative stress levels in hind limb suspension (HLS) mice. Concurrently, bone mineral density (BMD), bone microstructure, bone mechanical properties, and bone metabolism indicators were measured. In addition, the transcription levels of antioxidant enzyme- and osteogenic-related genes were measured to preliminarily uncover the mechanism by which LMWCSs alleviate oxidative stress and bone loss in simulated microgravity.

## 2. Materials and Methods

### 2.1. Materials

CS was purchased from Hefei Bomei Biotechnology Co., Ltd. (Cat. No. LC2215, Hefei, China). LMWCSs were derived from the degradation of CS following a previously published complex enzyme method [20]. The basic preparation process of LMWCSs was as follows: A complex enzyme solution containing a 6:4 Chondroitinase ABC I: Chondroitinase ABC II ratio with 0.1 U of total enzyme activity was added to a 7 g/L CS solution prepared in the buffer (50 mM Tris, 100 mM NaAc, pH 8.0). The mixture was incubated at 37 °C for 1 h, and the enzymatic reaction was terminated by boiling the mixture for 10 min. The enzymatic hydrolysis solution was collected via centrifugation at 10,000× *g* for 20 min. The solution was successively passed through ultrafiltration membranes with molecular weight cutoffs of 5 and 0.5 kDa (Millipore Corp., Bedford, MA, USA). The ultrafiltered components (0.5–5 kDa) were collected and freeze-dried to obtain LMWCSs.

### 2.2. Ethics Approval

All animal experiments in this study were approved by the Animal Ethical and Welfare Committee (NKYY-DWLL-2020-167) and complied with the principles of animal protection, animal welfare and ethics, and the relevant regulations of the National Laboratory Animal Welfare Ethics.

### 2.3. Animals

Male C57 mice who were 2 months old, weighing 23–26 g, were purchased from Beijing Huafukang Biotechnology Co., Ltd. (Beijing, China). All mice were maintained at a temperature of 23 ± 2 °C, under a 12/12 h light/dark cycle (light, 08:00–20:00; dark, 20:00–08:00) with a humidity of 45–50%. They had ad libitum access to standard laboratory food and water.

### 2.4. Grouping and Treatment of Animals

All mice were reared accordingly for 1 week and then randomly divided into 4 groups (6 mice per group): the non-HLS control group (Ctrl), the HLS model group (HLS), the HLS group fed CS (HLS + CS), and the HLS group fed LMWCSs (HLS + LMWCSs).

Except for the Ctrl group, all experimental groups were subjected to HLS with reference to the modified method of Wronski et al. [21]. Briefly, the tails of the mice were fixed to the metal rod on the top of the hindlimb suspension cage via a stainless steel ring. The forelimbs of the mice could touch the bottom of the cage, and the hindlimbs hung just off the horizontal plane, and the body formed an angle of 30°–40° with the horizontal plane so that the hindlimbs of the mice could not touch any supporting surface, but the mice could still move and access food and water freely. The mice were suspended for a total of 6 weeks. While the hindlimbs were suspended, LMWCS solutions at 180 mg/kg/d were administered to the mice in the HLS + LMWCSs group, while the HLS + CS group was treated with a CS solution at 180 mg/kg/d. The Ctrl and HLS groups were administered an equivalent volume of distilled water. Dose selection was based on the study that was previously conducted [18]. Mice in each group were administered the solutions daily for 6 weeks, during which time they ate and drank freely, were weighed once every 7 days, and the gavage dose was adjusted according to any changes in body weight. After 6 weeks, samples were taken to measure indicators related to oxidative stress, bone metabolism, and bone loss.

### 2.5. Collection and Preparation of Tissue Samples

#### 2.5.1. Collection of Blood Samples and Preparation of Serum

After the last gavage, the mice were subjected to a 12 h fast with access to water. Mice were anesthetized with isoflurane gas the following day, and whole blood was collected from the eye canthus. Whole blood was incubated at 20–25 °C for 10–20 min, centrifuged at 1000× *g* for 10 min, and the serum obtained was divided into sterile tubes and stored at −80 °C before analysis.

#### 2.5.2. Collection and Treatment of the Femur and Tibia

After the mice were euthanized, the muscles and tendons attached to the femur and tibia were removed and rinsed with normal saline. Following this, the right femur was wrapped in gauze soaked with normal saline and stored at −20 °C before bone mechanics. The tibia and the left femur were wrapped with medical gauze and stored in liquid nitrogen. The tibia was used for microcomputed tomography (micro-CT) and quantitative real-time polymerase chain reaction (qPCR). The left femur was crushed into powder under liquid nitrogen and homogenized using PBS (1:9, *w*/*v*) at 4 °C to adequately release the antioxidants therein. Subsequently, bone tissue homogenate was centrifuged at 4 °C, 12,000× *g* for 10 min, and the supernatant was taken for the oxidative/antioxidant indices determination.

#### 2.5.3. Urine Collection

After fasting, the mice were transferred to special metabolic cages for separate feeding, and their urine was collected for a period of 24 h. Following this, the urine was placed into sterile centrifuge tubes, centrifuged at 1500× *g* for 20 min, and the supernatant was collected for analysis.

### 2.6. Determination of the Oxidative/Antioxidant Indices in the Serum and Bone Tissue

The malondialdehyde (MDA) levels, total antioxidant capacity (T-AOC) values, superoxide dismutase (SOD) activity, and glutathione (GSH) levels in the serum and bone tissue of mice were assessed using assay kits according to the manufacturer’s instructions. The MDA and T-AOC assay kits were manufactured by Beyotime Institute of Biotechnology (Cat. No. were S0131 and S0116, respectively, Shanghai, China). The SOD and GSH assay kits were both manufactured at the Nanjing Jiancheng Bioengineering Research Institute (Cat. No. were A001-3 and A006-2-1, respectively, Nanjing, China). The levels of MDA, T-AOC, and GSH and SOD activity in unit volume serum and unit weight bone tissue were calculated according to the equation in the kit instructions.

### 2.7. Micro-CT Analysis

The right tibias of the mice were scanned using a SkyScan 1174 X-ray microtomography system (Bruker, Kontich, Belgium), with the following experimental parameters: operating voltage set at 50 kV, electric current of 800 µA, spatial resolution of 9.76 µm, tomographic image resolution of 1304 × 1024 pixels, and rotation step of 0.6°. After scanning, a 3-dimensional (3D) image of the sample was constructed through the collected 2-dimensional slices (2D) using N-Recon software (Bruker, Kontich, Belgium). CT-AN 3-dimensional morphometry software (Bruker, Kontich, Belgium) was used to analyze the 3D reconstructed images of the region of interest (ROI). The ROI for cancellous bone was located 0.6 mm below the growth plate of the proximal tibia and continued distally for 100 slices, with a thickness of 1.2 mm. The following parameters were obtained by analyzing the ROI for cancellous bone: cancellous BMD, trabecular bone volume fraction (BVF), trabecular bone surface to bone volume (BS/BV), trabecular number (Tb. N), trabecular thickness (Tb. Th), and trabecular spacing (Tb. Sp). Cortical bone was analyzed by a region of 100 slices, starting 6 mm distal to the ROI for the trabecular bone, with a thickness of 1.2 mm. Cortical parameters included cortical BMD and cortical thickness (Ct. Th).

### 2.8. Bone Mechanical Properties Test

In this experiment, a dynamic mechanical analyzer (Bose ElectroForce 3230, Bose Corp., ElectroForce Systems Group, Eden Prairie, MN, USA) was used to assess the mechanical properties of bone using the 3-point bending method. Twelve hours before the test, the right femur samples were thawed at 20–25 °C and re-humidified with normal saline. The loading point was located in the middle of the measured femoral shaft, with the distance between the 2 supporting points being 10 mm. During the test, a constant speed of 0.16 mm/s was applied to the bone until it broke, and 20 load–displacement data were collected every second. The load–displacement curve was generated from these data, and the ultimate force (N) was calculated, being the peak of the curve on the *Y*-axis. Stiffness (the slope of the linear part of the load–displacement curve, N/mm) and maximum absorption energy (the area between the vertical and horizontal axes at the limit load point of the curve, mJ) were also determined.

### 2.9. Determination of Biochemical Indicators of Bone Metabolism

#### 2.9.1. Determination of Alkaline Phosphatase (ALP) Activity and Osteocalcin (OCN) Levels

ALP activity and OCN levels in serum were detected using an alkaline phosphatase test kit and a mouse OCN ELISA kit, respectively. Both kits were purchased from Nanjing Jiancheng Bioengineering Research Institute (Cat. No. were A059-2 and H152, respectively, Nanjing, China) and were used following the manufacturer’s instructions. The seral ALP activity and OCN levels were normalized to U/100 mL serum and ng/mL serum, respectively.

#### 2.9.2. Determination of Deoxypyridinoline (DPD) and Creatinine (CRE)

Urine DPD levels were determined using a mouse DPD ELISA kit (Cat. No. H291-1-2, Nanjing Jiancheng Bioengineering Research Institute, Nanjing, China). CRE levels were determined using a CRE assay kit (Cat. No. C011-2-1, Nanjing Jiancheng Bioengineering Research Institute, Nanjing, China) according to the manufacturer’s instructions. Urine DPD levels were adjusted and expressed as the ratio of DPD to CRE (DPD/CRE) to eliminate the effect of urine concentration and dilution.

### 2.10. qPCR Detection of mRNA Levels

TRIzol reagent (Cat. No. 15596-018, Thermo Fisher, Scientific, Rockford, IL, USA) was used to extract total RNA from the right tibia. cDNA was prepared using a reverse transcription kit (Cat. No. A5001, Promega Corp., Madison, WI, USA) following the manufacturer’s instructions. qPCR was conducted using a SYBR^®^ Green Realtime PCR Master Mix Kit (Cat. No. QPK-201, Toyobo, Japan). PCR amplification was performed using the Stratagene Mx3000 quantitative PCR system (Agilent Technologies, Santa Clara, CA, USA) and carried out in a 96-well optical reaction plate for 40 cycles of 95 °C for 10 s, 60 °C for 60 s, and 72 °C for 30 s. The mRNA expression levels of NF-E2-related factor 2 (*Nrf2*), quinone oxidoreductase1 (*NQO1*), *ALP*, *OCN*, and runt-related transcription factor 2 (*Runx2*) were assessed using primers with the sequences shown in Table S1 (see in Supplementary Materials). All primers were procured by Suzhou Jinweizhi Biotechnology Co., Ltd. (Suzhou, China). The relative levels of mRNA were normalized to those of glyceraldehyde 3-phosphate dehydrogenase (*GAPDH*), and the 2^−∆∆ct^ method [22] was used to determine the relative expression levels of each gene.

### 2.11. Statistical Analysis

Statistical analysis was performed using SPSS 22.0 statistical software (IBM Co., Armonk, NY, USA). All data were tested for normal distribution and homogeneity of variance before data analysis. Data in conformity with the normality and homogeneity of variances were analyzed by the one-way analysis of variance (ANOVA) with Tukey’s post hoc multiple comparisons. One-way ANOVA with Dunnett’s T3 multiple test was used when the variance was non-homogeneous, but in accordance with the normality. Data are expressed as mean ± standard deviation (SD), and *p*-values < 0.05 were considered statistically significant.

## 3. Results

### 3.1. The General Effect of LMWCSs Treatment in Mice

As shown in Figure S1 (see in Supplementary Materials), there was no significant difference in the initial body weights among all groups. After administration for 6 weeks, a minor gain in body weight was observed in all groups. The weight gain was relatively higher in the Ctrl and HLS + LMWCSs groups, but their final body weights did not significantly differ among all groups.

### 3.2. LMWCSs abate Oxidative Stress in HLS Mice

**Figure 1:**
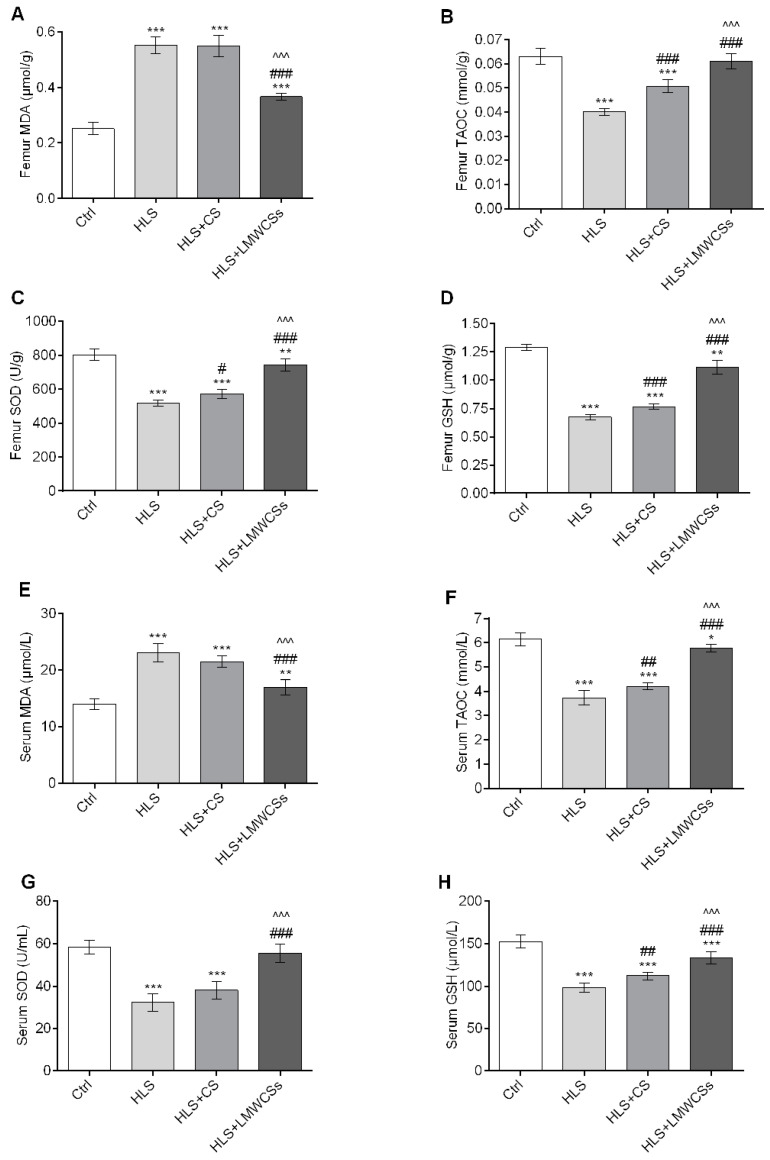
Effects of low-molecular-weight chondroitin sulfates (LMWCSs) on oxidative/antioxidant indices in serum and femoral tissues of hindlimb suspension mice (*n* = 6 per group). (**A**) Comparison of femur malondialdehyde (MDA) levels in mice in each group. (**B**) Comparison of femur total antioxidant capacity (T-AOC) values in mice in each group. (**C**) Comparison of femur superoxide dismutase (SOD) activities in mice in each group. (**D**) Comparison of femur glutathione (GSH) levels in mice in each group. (**E**) Comparison of serum MDA levels in mice in each group. (**F**) Comparison of serum T-AOC values in mice in each group. (**G**) Comparison of serum SOD activities in mice in each group. (**H**) Comparison of serum GSH levels in mice in each group. Data in panels (**A**–**H**) were analyzed by one-way ANOVA with Tukey’s post hoc multiple comparisons test. Data in panel D were analyzed by one-way ANOVA with Dunnett’s T3 post hoc multiple comparisons test. * *p* < 0.05 vs. Ctrl group; ^#^ *p* < 0.05 vs. HLS group; ** *p* < 0.01 vs. Ctrl group; ^##^ *p* < 0.01 vs. HLS group; *** *p* < 0.001 vs. Ctrl group; ^###^ *p* < 0.001 vs. HLS group; ^^^^^ *p* < 0.001 vs. HLS + CS group. Ctrl: control group; HLS: hindlimb suspension group; HLS + CS: hindlimb suspension and treated with chondroitin sulfate group; HLS + LMWCSs: hindlimb suspension and treated with low-molecular-weight chondroitin sulfates group.

Compared with the Ctrl group, the femoral MDA level (Figure 1A) in the HLS group increased significantly (F(3, 20) = 168.168, *p* < 0.001), and the femoral T-AOC value (Figure 1B), SOD activity (Figure 1C), and GSH level (Figure 1D) significantly decreased (F(3, 20) = 88.063, 133.189, and 357.090 for T-AOC, SOD, and GSH, respectively; all *p* < 0.001). However, compared with the HLS group, these four indicators related to oxidative stress were significantly reversed in the HLS + LMWCSs group (*p* < 0.001, *p* < 0.001, *p* = 0.008, and *p* = 0.002, respectively; Figure 1A–D). CS treatment could also reduce the decrease in the femoral T-AOC value, SOD activity, and GSH level after HLS treatment (Figure 1B–D), but its effect was weaker than that of LMWCSs. Changes in the serum MDA level, T-AOC value, SOD activity, and GSH level in each group were basically consistent with those observed in the femur (Figure 1E–H).

The above results showed that exposure to HLS led to oxidative stress in bone tissue and the circulatory system of mice. Treatment with LMWCSs could effectively reduce these oxidative stresses, and their overall effect was better than that of CS.

### 3.3. LMWCSs Increase the Antioxidant Capacity through Regulation of the mRNA Expression Levels of Antioxidant Enzyme-Related Genes in HLS Mice

**Figure 2:**
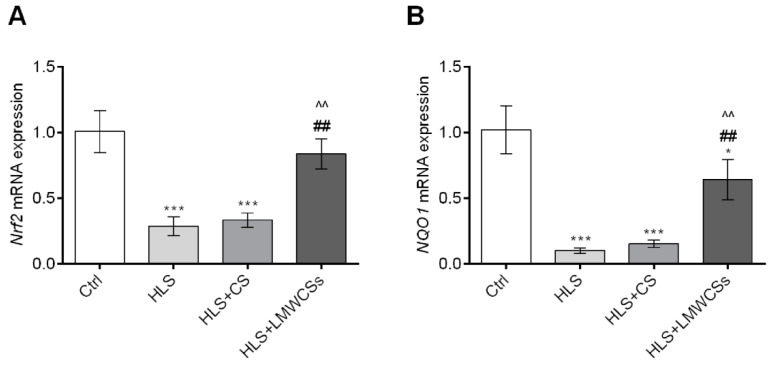
Effects of low-molecular-weight chondroitin sulfates (LMWCSs) on mRNA expression levels of antioxidant enzyme-related genes in hindlimb suspension mice (*n* = 3 per group). (**A**) mRNA expression levels of NF-E2-related factor 2 (*Nrf2*) in the tibia of mice in each group. (**B**) mRNA expression levels of quinone oxidoreductase1 (*NQO1*) in the tibia of mice in each group. Data were analyzed by one-way ANOVA with Tukey’s post hoc multiple comparisons test. * *p* < 0.05 vs. Ctrl group; ^##^ *p* < 0.01 vs. HLS group; ^^^^ *p* < 0.01 vs. HLS + CS group; *** *p* < 0.001 vs. Ctrl group. Ctrl: control group; HLS: hindlimb suspension group; HLS + CS: hindlimb suspension and treated with chondroitin sulfate group; HLS + LMWCSs: hindlimb suspension and treated with low-molecular-weight chondroitin sulfates group.

As shown in Figure 2A,B, the tibial mRNA expression levels of the antioxidant enzyme-related gene *Nrf2* and its downstream gene *NQO1* in the HLS group were all dramatically downregulated compared with those in the Ctrl group (F(3, 8) = 33.101 and 39.199 for *Nrf2* and *NQO1*, respectively; both *p* < 0.001). However, LMWCSs treatment significantly upregulated the transcriptional levels of *Nrf2* and *NQO1* in HLS mice (*p* = 0.001 and *p* = 0.003, respectively). Additionally, there was no significant difference in *Nrf2* and *NQO1* expression between the CS group and the HLS group. These results indicate that LMWCSs could significantly reduce the oxidative stress which occurs in the bone tissue of HLS mice by regulating the mRNA expression levels of *Nrf2* and *NQO1*. The regulation effect of CS was not obvious.

### 3.4. LMWCSs Treatment Alleviates Bone Loss Induced by HLS in Mice

To explore the effects of LMWCSs on the changes in bone mass, microstructure, and mechanical strength induced by simulated microgravity, bone micro-CT analysis and the mechanical properties test in HLS mice were performed.

**Figure 3:**
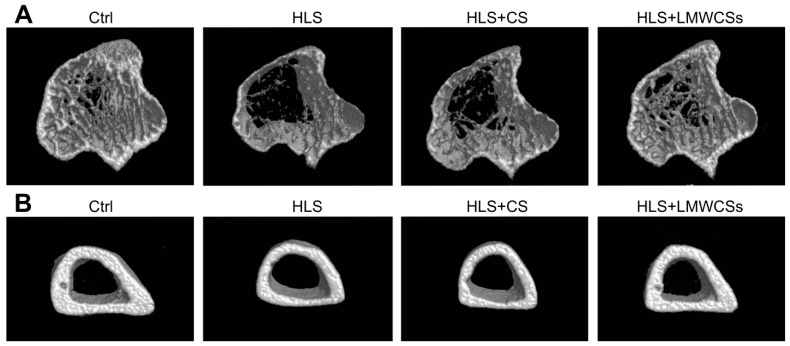
Representative microcomputed tomography (micro-CT) reconstructive images of trabecular and cortical bone in various treatment groups. (**A**) Representative micro-CT reconstructive images of the trabecular bone in various treatment groups. (**B**) Representative micro-CT reconstructive images of the cortical bone in various treatment groups. Ctrl: control group; HLS: hindlimb suspension group; HLS + CS: hindlimb suspension and treated with chondroitin sulfate group; HLS + LMWCSs: hindlimb suspension and treated with low-molecular-weight chondroitin sulfates group.

Representative 3D images of trabecular and cortical bone are shown in Figure 3. The analysis results of cancellous and cortical bone parameters by micro-CT are presented in Table 1. Compared with the Ctrl group, HLS treatment resulted in a significant decrease in cancellous BMD (F(3, 8) = 45.301, *p* < 0.001), trabecular BVF (F(3, 8) = 55.887, *p* < 0.001), thickness (Tb.Th) (F(3, 8) = 25.671, *p* < 0.00), and number (Tb.N) (F(3, 8) = 28.088, *p* < 0.001), with a concomitant obvious increase in the trabecular bone surface to bone volume (BS/BV) (F(3, 8) = 34.387, *p* < 0.001). LMWCSs treatment alleviated the alteration of cancellous bone parameters induced by HLS in mice. Cancellous BMD (*p* < 0.001), trabecular BVF (*p* = 0.002), Tb. Th (*p* = 0.012), and Tb. N (*p* = 0.011) were significantly improved by LMWCSs treatment, while BS/BV (*p* = 0.003) were significantly reduced compared with HLS mice. In addition, mice in the HLS group showed thinner cortices and lower cortical BMD than the Ctrl group, whereas LMWCSs revealed a prevention effect on the cortical BMD in the tibia of HLS mice. Compared with LMWCSs, CS had less of an effect on the micro-architecture of cancellous and cortical bone. As shown in Table 1, the cancellous and cortical bone parameters in the HLS + CS group were not significantly different from those in the HLS group.

**Figure 4:**
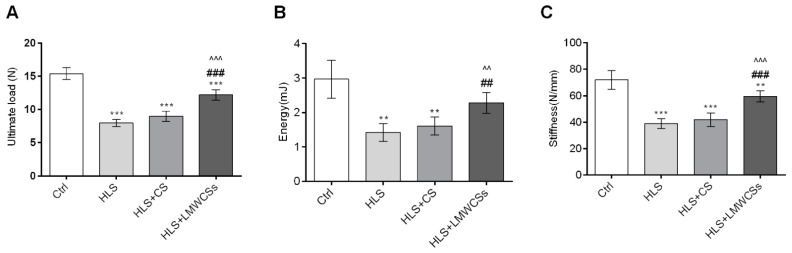
Effects of low-molecular-weight chondroitin sulfates (LMWCSs) on mechanical properties of hindlimb suspension mice (*n* = 6 per group). (**A**) Mean ultimate load of mice femurs from each group. (**B**) Mean maximum energy absorption of mice femurs from each group. (**C**) Mean femur stiffness in mice from each group. Data were analyzed by one-way ANOVA with Tukey’s post hoc multiple comparisons test. ** *p* < 0.01 vs. Ctrl group; ^##^ *p* < 0.01 vs. HLS group; ^^^^ *p* < 0.01 vs. HLS + CS group; *** *p* < 0.001 vs. Ctrl group; ^###^ *p* < 0.001 vs. HLS group; ^^^^^ *p* < 0.001 vs. HLS + CS group. Ctrl: control group; HLS: hindlimb suspension group; HLS + CS: hindlimb suspension and treated with chondroitin sulfate group; HLS + LMWCSs: hindlimb suspension and treated with low-molecular-weight chondroitin sulfates group.

The results of the bone mechanical properties test on the femur are shown in Figure 4; the femoral ultimate load (Figure 4A), maximum absorption energy (Figure 4B), and stiffness (Figure 4C) in the HLS group were all significantly reduced compared with those in the Ctrl group (F(3, 20) = 118.907, 22.134, and 53.502 for ultimate load, energy, and stiffness, respectively; *p* < 0.001, *p* = 0.002, and *p*< 0.001, respectively). The ultimate load, maximum absorption energy, and stiffness in the HLS + LMWCSs group were significantly higher than those in the HLS group (*p* < 0.001, *p* = 0.002, and *p*< 0.001, respectively) and they increased by 52.8%, 60.7%, and 52.9%, respectively. However, these three mechanical parameters in the HLS + CS group were not significantly different from those in the HLS group.

Taken together, these results indicate that exposure to HLS resulted in significant bone mass loss and alteration in bone microstructure and mechanical strength in mice. Treatment with LMWCSs alleviated these bone damages caused by simulated microgravity in HLS mice.

### 3.5. LMWCSs Regulate the Imbalance of Bone Metabolism in HLS Mice

**Figure 5:**
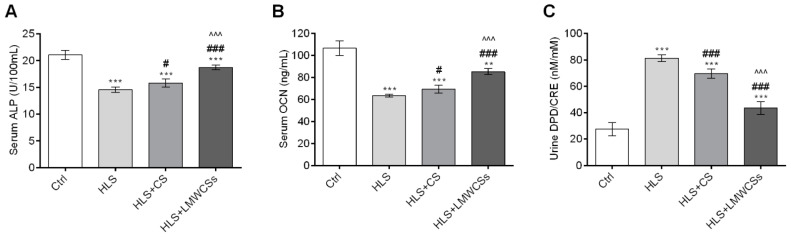
Effects of low-molecular-weight chondroitin sulfates (LMWCSs) on biochemical indices of bone metabolism in hindlimb suspension mice (*n* = 6 per group). (**A**) Mean serum alkaline phosphatase (ALP) activity in mice from each group. (**B**) Mean serum osteocalcin (OCN) levels in mice from each group. (**C**) Mean urine deoxypyridinoline/creatinine (DPD/CRE) ratio of mice in each group. Data in panels (**A**,**C**) were analyzed by one-way ANOVA with Tukey’s post hoc multiple comparisons test. Data in panel B were analyzed by one-way ANOVA with Dunnett’s T3 post hoc multiple comparisons test. ^#^ *p* < 0.05 vs. HLS group; ** *p* < 0.01 vs. Ctrl group; *** *p* < 0.001 vs. Ctrl group; ^###^ *p* < 0.001 vs. HLS group; ^^^^^ *p* < 0.001 vs. HLS + CS group. Ctrl: control group; HLS: hindlimb suspension group; HLS + CS: hindlimb suspension and treated with chondroitin sulfate group; HLS + LMWCSs: hindlimb suspension and treated with low-molecular-weight chondroitin sulfates group.

As shown in Figure 5A,B, the serum ALP activity and OCN level were significantly reduced (F(3, 20) = 119.586 and 135.963 for ALP and OCN, respectively; both *p* < 0.001) in the HLS group compared with those in the Ctrl group, whereas after LMWCSs were administered, the levels of these two osteogenic markers increased by 28.6% and 34.2%, respectively (both *p* < 0.001). On the contrary, the urine DPD/CRE ratio (Figure 5C), a bone resorption biochemical index, was significantly increased (F(3, 20) = 213.424, *p* < 0.001) in HLS mice compared with that in Ctrl mice and decreased by 46.5% when administered with LMWCSs compared with that in the HLS group (*p* < 0.001, Figure 5C). In addition, CS treatment could also obviously restore the decrease of the serum ALP activity (*p* = 0.02, Figure 5A) and reduced the increase of the urine DPD/CRE ratio in HLS mice (*p* < 0.001, Figure 5C), but its effect was significantly weaker than that of LMWCSs (both *p* < 0.001). These results showed that LMWCSs could regulate the imbalance of bone metabolism caused by simulated microgravity by enhancing bone formation and inhibiting bone resorption in HLS mice, and their effect was better than that of CS.

### 3.6. LMWCSs Improve Osteoblast Activity through Regulation of the mRNA Expression Levels of Osteogenic-Related Genes in HLS Mice

**Figure 6:**
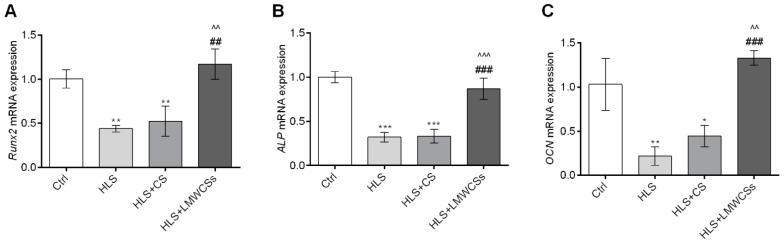
Effects of low-molecular-weight chondroitin sulfates (LMWCSs) on mRNA expression levels of osteogenic marker-related genes in hindlimb suspension mice (*n* = 3 per group). (**A**) mRNA expression levels of runt-related transcription factor 2 (*Runx2*) in the tibia of mice in each group. (**B**) mRNA expression levels of alkaline phosphatase (ALP) in the tibia of mice in each group. (**C**) mRNA expression levels of osteocalcin (*OCN*) in the tibia of mice in each group. Data were analyzed by one-way ANOVA with Tukey’s post hoc multiple comparisons test. * *p* < 0.05 vs. Ctrl group; ** *p* < 0.01 vs. Ctrl group; ^##^ *p* < 0.01 vs. HLS group; ^^^^ *p* < 0.01 vs. HLS + CS group; *** *p* < 0.001 vs. Ctrl group; ^###^ *p* < 0.001 vs. HLS group; ^^^^^ *p* < 0.001 vs. HLS + CS group. Ctrl: control group; HLS: hindlimb suspension group; HLS + CS: hindlimb suspension and treated with chondroitin sulfate group; HLS + LMWCSs: hindlimb suspension and treated with low-molecular-weight chondroitin sulfates group.

Compared with the Ctrl group, after HLS treatment, the tibia mRNA expression levels of the osteogenic-related genes *Runx2* (Figure 6A), *ALP* (Figure 6B), and *OCN* (Figure 6C) were all significantly downregulated (F(3, 8) = 21.715, 54.254, and 26.572 for *Runx2*, *ALP* and *OCN,* respectively; *p* = 0.004, *p* < 0.001, and *p* = 0.002, respectively). Compared with the HLS group, mRNA expression levels of these three genes were all significantly increased upon LMWCSs treatment (*p* = 0.001, *p* < 0.001, and *p* < 0.001, respectively), whereas the difference was not significant between the CS group and the HLS groups. These results indicated that LMWCSs could facilitate the osteoblast activity in the HLS mice better than CS.

## 4. Discussion

In order to examine whether LMWCSs have good antioxidant activity in vivo and explore whether they can play a beneficial role in oxidative stress injury due to simulated microgravity in mice, we explored the effects of LMWCSs on bone tissue and serum MDA content, reduced GSH content, T-AOC, SOD activity, and other oxidation/antioxidant indices, as well as the transcription levels of bone tissue antioxidant enzyme-related genes in HLS mice. MDA is the final product of lipid peroxidation and is a commonly used biomarker to assess oxidative stress [23]. MDA levels in the bone tissues and sera of HLS mice increased significantly, whereas T-AOC values significantly decreased, further confirming that HLS mice may be in a state of oxidative stress induced by excessive reactive oxygen species (ROS). GSH is an important antioxidant in the human body, which can eliminate ROS and thus prevent ROS-induced damage to the body [24]. In the present study, GSH levels in the bone tissues and sera of HLS mice significantly decreased, indirectly suggesting that the production of ROS increased. In addition, reduced antioxidant enzyme activity could also lead to increased oxidative stress. Here, SOD activity and mRNA expression levels of antioxidant enzyme-related genes (*Nrf2* and *NQO1*) were assessed. The results showed that the activity of the antioxidant enzyme defense system in the sera and bone tissues of HLS mice decreased, which further confirmed that HLS mice were in a state of oxidative stress. The above experimental results further proved the research viewpoint that microgravity can induce oxidative stress in bone tissue [1,25].

However, after HLS mice were fed with LMWCSs for 6 weeks, MDA content in the bone tissue and serum decreased significantly, while the GSH content, T-AOC level, SOD activity, and mRNA expression levels of antioxidant enzyme-related genes increased significantly, suggesting that LMWCSs had good antioxidant activity in vivo and could reverse oxidative stress damage caused by simulated microgravity in mice.

Recent studies showed that oxidative stress is clearly involved in the occurrence and development of osteoporosis [26]. Oxidative stress can induce osteoblast apoptosis and inhibit their proliferation and differentiation [27,28], which may be an important mechanism of bone loss in microgravity [29]. Therefore, in this study, while exploring the effects of LMWCSs on oxidative/antioxidant indices of serum and bone tissue in HLS mice, the effects of LMWCSs on BMD, bone mechanical properties, and the bone microstructure of HLS mice were determined. The results showed that LMWCSs treatment significantly improved the BMD of HLS mice and their bone mechanical properties and bone microstructure, suggesting that LMWCSs had a good potential for alleviating bone loss under simulated microgravity.

Bone metabolism indices can effectively reflect the activity of osteoblasts and osteoclasts, thus revealing changes in the dynamic balance of bone formation and bone resorption [30,31,32]. The results of bone metabolism indices in this study showed that LMWCSs could increase serum ALP activity and OCN content, and reduce the urine DPD/CRE level, suggesting that LMWCSs could promote bone formation and inhibit bone resorption in microgravity. The results of the mRNA expression levels of bone formation-related genes (*Runx2*, *ALP*, and *OCN*) indicated that LMWCSs could improve the activity of osteoblasts in HLS mice by regulating the mRNA expression levels of bone formation related genes.

The above experimental results indicate that oxidative stress injury in HLS mice was prevented, and bone loss was diminished after LMWCSs administration, which confirms our hypothesis that LMWCSs not only have good antioxidant activity in vivo, but they may diminish the bone loss induced by simulated microgravity in mice with antioxidant effects. An increasing number of studies have shown that the appropriate intake of antioxidants can effectively alleviate bone loss caused by microgravity. For example, curcumin isolated from the roots of turmeric and polyphenols isolated from the conical scales of red pine reduce HLS-induced bone loss in rats by inhibiting oxidative stress [5,33].

Current studies suggest that a major mechanism of cellular defense against oxidative stress is the activation of the Nrf2 signaling pathway with Nrf2 at its core [34]. Activation of the Nrf2 signaling pathway can effectively remove free radicals generated in the body and regulate the expression of various antioxidant enzymes including NQO1 and heme oxygenase-1 [35]. In addition, Wang et al. [36] found that activation of the Nrf2 signaling pathway mitigates the decrease in osteoblast activity under simulated microgravity. To investigate the mechanism of LMWCSs in alleviating oxidative stress and bone loss in simulated microgravity, the mRNA expression levels of *Nrf2* and its downstream gene, *NQO1*, were detected in this study. We found that LMWCSs significantly prevented HLS-induced downregulation of *Nrf2* and *NQO1* mRNA expression in bone tissue (*p* < 0.01), suggesting that LMWCSs may contribute to the activation of the Nrf2 signaling pathway, thus reducing the oxidative stress level in bone tissue under simulated microgravity, protecting osteoblast activity, and mitigating bone loss caused by simulated microgravity.

## 5. Conclusions

In conclusion, LMWCSs prepared from CS using a safe and environmentally friendly complex enzyme method have good antioxidant activity in vivo and may significantly reduce the oxidative damage and bone loss caused by simulated microgravity. Therefore, LMWCSs can be envisaged as potential antioxidants and bone loss protective agents in microgravity environments. The results of this study have potential significance for the further development and utilization of LMWCSs, as well as for treating bone loss caused by microgravity or oxidative stress experienced by astronauts and long-term bedridden patients. However, further research is needed to reveal the mechanisms of anti-oxidative stress and anti-bone loss of LMWCSs in vivo. Additionally, more studies regarding dosage, treatment duration, and administration mode of LMWCSs will also be crucial before clinical application will be possible.

## Figures and Tables

**Table 1:** The cancellous and cortical bone parameters of tibias in various treatment groups.

	Ctrl	HLS	HLS + CS	HLS + LMWCSs
Cancellous bone				
BMD (g/cm^3^)	0.121 ± 0.017	0.041 ± 0.003 ***	0.049 ± 0.004 ***	0.082 ± 0.006 ***^###^^^^
BVF (%)	10.84 ± 1.77	1.34 ± 0.34 ***	1.73 ± 0.12 ***	6.21 ± 1.01 **^##^^^
BS/BV (%)	69.36 ± 2.09	111.45 ± 7.70 ***	103.61 ± 6.00 ***	86.93 ± 4.72 *^##^^
Tb. Th (mm)	0.060 ± 0.003	0.042 ± 0.002 ***	0.045 ± 0.003 **	0.051 ± 0.003 *^#^
Tb. N (1/mm)	1.831 ± 0.373	0.321 ± 0.082 ***	0.388 ± 0.009 ***	1.143 ± 0.266 *^#^^
Tb. Sp (mm)	0.271 ± 0.024	0.479 ± 0.081	0.481 ± 0.079	0.304 ± 0.023
Cortical bone				
BMD (g/cm^3^)	1.407 ± 0.011	1.359 ± 0.005 *	1.343 ± 0.033	1.402 ± 0.011 ^#^^
Ct. Th (mm)	0.247 ± 0.018	0.183 ± 0.016 **	0.191 ± 0.009 **	0.205 ± 0.014 *

Data are expressed as mean ± SD, *n* = 3 in each group. Data (cancellous BMD, BVF, BS/BV, Tb. Th, Tb. N, and Ct. Th) were analyzed by one-way ANOVA with Tukey’s post hoc multiple comparisons test. Data (Tb. Sp and Cortical BMD) were analyzed by one-way ANOVA with Dunnett’s T3 post hoc multiple comparisons test. * *p* < 0.05 vs. Ctrl group; ^#^ *p* < 0.05 vs. HLS group; ^^^ *p* < 0.05 vs. HLS + CS group; ** *p* < 0.01 vs. Ctrl group; ^##^ *p* < 0.01 vs. HLS group; ^^^^ *p* < 0.01 vs. HLS + CS group; *** *p* < 0.001 vs. Ctrl group; ^###^ *p* < 0.001 vs. HLS group; ^^^^^ *p* < 0.001 vs. HLS + CS group. Ctrl: control group; HLS: hindlimb suspension group; HLS + CS: hindlimb suspension and treated with chondroitin sulfate group; HLS + LMWCSs: hindlimb suspension and treated with low-molecular-weight chondroitin sulfates group.

## Data Availability

Data sharing not applicable.

## Supplementary Materials

The following supporting information can be downloaded at: https://www.mdpi.com/article/10.3390/cimb45050268/s1, Table S1: Sequences of primers used for qPCR; Figure S1: Body weight changes in all groups (*n* = 6 per group).

## Abbreviations

ALP: alkaline phosphatase; BMD: bone mineral density; BS/BV: bone surface to bone volume; BVF: bone volume fraction; CRE: creatinine; CS: chondroitin sulfate; Ct. Th: cortical thickness; DPD: deoxypyridinoline; GAPDH: glyceraldehyde 3-phosphate dehydrogenase; GSH: glutathione HLS: hind limb suspension; LMWCSs: low-molecular-weight chondroitin sulfates; MDA: malondialdehyde; micro-CT: microcomputed tomography; NQO1: quinone oxidoreductase 1; Nrf2: NF-E2-related factor 2; OCN: osteocalcin; qPCR: quantitative real-time polymerase chain reaction; ROI: region of interest; ROS: reactive oxygen species; Runx2: runt-related transcription factor 2; SOD: superoxide dismutase; T-AOC: total antioxidant capacity; Tb. N: trabecular number; Tb. Sp: trabecular spacing; Tb. Th: trabecular thickness.
