# Supplementary material for: Low-Molecular-Weight Chondroitin Sulfates Alleviate Simulated Microgravity-Induced Oxidative Stress and Bone Loss in Mice
Source: Curr Issues Mol Biol. 2023 May 10;45(5):4214–27. doi: 10.3390/cimb45050268 (PMC10217679; doi:10.3390/cimb45050268)
Supplement: Supplementary file 1 [file cimb-45-00268-s001.zip › cimb-2259286-supplementary.pdf]

Supplementary materials

Table S1. Sequences of primers used for qPCR

Genes	Primer sequences (5′-3′)
**GADPH (mouse)**	Forward: AGAAGGTGGTGAAGCAGGCATCT
	Reverse: CGGCATCGAAGGTGGAAGAGTG
**OCN**	Forward: TTCTGCTCACTCTGCTGACC
	Reverse: GCCGGAGTCTGTTCACTACC
**Runx2**	Forward: TTCCAGACCAGCAGCACTCCAT
	Reverse: TTCCATCAGCGTCAACACCATCATT
**ALP**	Forward: GGCTACTTCTTCGTGGAG
	Reverse: GCGTGAGTGTTCTTGTCT
**NQO1**	Forward: AGCCAATCAGCGTTCGGTAT
	Reverse: GCCTCCTTCATGGCGTAGTT
**Nrf2**	Forward: AACAGAACGGCCCTAAAGCA
	Reverse: TGGGATTCACGCATAGGAGC

Abbreviations: qPCR, quantitative real-time polymerase chain reaction; GAPDH, glyceraldehyde-3-phosphate dehydrogenase; OCN, osteocalcin; Runx2, runt-related transcription factor 2; ALP, alkaline phosphatase; NQO1, quinone oxidoreductase 1; Nrf2, NF-E2-related factor 2.

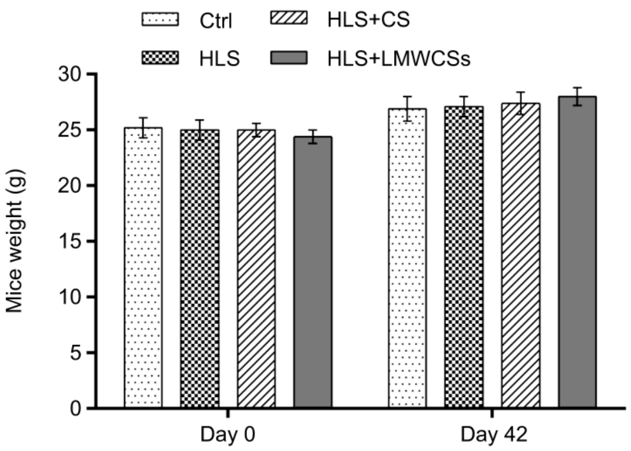

Figure S1. Body weight changes in all groups (n=6 per group)

Ctrl: control group; HLS: hindlimb suspension group; HLS + CS: hindlimb suspension and treated with chondroitin sulfate group; HLS + LMWCSs: hindlimb suspension and treated with low-molecular-weight chondroitin sulfates group.
